# The premarket assessment of the cost-effectiveness of a predictive technology “Straticyte™” for the early detection of oral cancer: a decision analytic model

**DOI:** 10.1186/s13561-017-0170-6

**Published:** 2017-10-02

**Authors:** S. Khoudigian-Sinani, G. Blackhouse, M. Levine, L. Thabane, D. O’Reilly

**Affiliations:** 1 Department of Health Research, Methods, Evidence and Impact, Faculty of Health Sciences, McMaster University, Hamilton, ON Canada; 2 PATH Research Institute, St Joseph’s Healthcare Hamilton, Hamilton, ON Canada; 3 Research Institute of St. Joseph’s, Hamilton, ON Canada; 4 Health Research Methodology (HRM), specializing in Health Technology Assessment (HTA), Hamilton, Canada; 5 Centre of Evaluation of Medicines, Father Sean O’Sullivan Research Centre, St. Joseph’s Healthcare Hamilton, Hamilton, Canada; 6 Patented Medicine Prices Review Board (Canada), Ottawa, Canada; 7 Department of Anesthesia/ Pediatrics, Faculty of Health Science, McMaster University, Hamilton, Canada; 8 Biostatistics Unit, St Joseph’s Healthcare, Hamilton, Canada; 9 Population Health Research Institute, Hamilton Health Sciences, Hamilton, Canada; 10 Programs for Assessment of Technology in Health (PATH) Research Institute, St. Joseph’s Healthcare, Hamilton, Canada; 11 Early Researcher Award Recipient, Ministry of Research and Innovation, Toronto, Canada

**Keywords:** Cost-effectiveness analysis, Early health technology assessment, Histopathology, Decision- analytic model, Early detection, Prognosis

## Abstract

**Introduction:**

Approximately half of oral cancers are detected in advanced stages. The current gold standard is histopathological assessment of biopsied tissue, which is subjective and dependent on expertise. *Straticyte™*, a novel prognostic tool at the pre-market stage, that more accurately identifies patients at high risk for oral cancer than histopathology alone. This study conducts an early cost-effectiveness analysis (CEA) of *Straticyte™* and histopathology versus histopathology alone for oral cancer diagnosis in adult patients.

**Methods:**

A decision-analytic model was constructed after narrowing the scope of *Straticyte™*, and defining application paths. Data was gathered using the belief elicitation method, and systematic review and meta-analysis. The early CEA was conducted from private-payer and patient perspectives, capturing both direct and indirect costs over a five-year time horizon. One-way and probabilistic sensitivity analyses were conducted to investigate uncertainty.

**Results:**

Compared to histopathology alone, histopathology with *Straticyte™* was the dominant strategy, resulting in fewer cancer cases (31 versus 36 per 100 patients) and lower total costs per cancer case avoided (3,360 versus 3,553). This remained robust when *Straticyte™* was applied to moderate and mild cases, but became slightly more expensive but still more effective than histopathology alone when *Straticyte™* was applied to only mild cases. The probabilistic and one-way sensitivity analyses demonstrated that incorporating *Straticyte™* to the current algorithm would be cost-effective over a wide range of parameters and willingness-to-pay values.

**Conclusion:**

This study demonstrates high probability that *Straticyte™* and histopathology will be cost-effective, which encourages continued investment in the product. The analysis is informed by limited clinical data on *Straticyte™*, however as more data becomes available, more precise estimates will be generated.

**Electronic supplementary material:**

The online version of this article (10.1186/s13561-017-0170-6) contains supplementary material, which is available to authorized users.

## Background

Economic evaluations (EEs) are increasingly used to inform decisions of healthcare resource allocation for interventions, including drugs and medical devices [1]. EEs, primarily cost-effectiveness analyses (CEA), are done for reimbursement in the late stages (i.e. post-market) of an intervention’s development. Reimbursement facilitates wide implementation in clinical practice, which improves return on investment and patients’ access to care. Recently, there has been interest in conducting early (i.e. pre-market) CEA, which gives companies feedback from content experts and stakeholders during their development and pre-market process [2]. Early CEA better prepares the company for licensing and adoption of the product, and may increase the likelihood of reimbursement by building a stronger evidence portfolio [2, 3]. Late CEA is a one-time process, whereas early CEA is iterative [4]. There are currently no guidelines in place on conducting early CEA, however several qualitative and quantitative approaches have been proposed (Additional file 1: Table S1).

Oral cancer encompasses cancers of the lip, oral cavity, or oropharynx, and accounts for 3% of all cancers worldwide [5, 6]. Though less common in Canada, 4100 new cases were estimated in 2013. The overall incidence in Canada is an estimated 12 cases per 100,000 people per year in men, and 5 per 100,000 in women [7, 8]. Up to 50% of oral cancers are not detected until the disease is well advanced and the overall survival rate, five years after diagnosis, is about 62% [6, 8]. Mortality can be reduced if treatment is initiated at an early stage, thus early diagnosis is critical.

The current gold standard for diagnosis is histopathologic assessment of a tissue biopsy, which is subjective. *Straticyte™*, a biomarker, is a novel prognostic tool for oral cancer. Based on an evaluation of 107 cases of dysplasia, with up to 10 years of follow-up, *Straticyte™* and histopathology demonstrated improvement in both the positive predicted value (PPV) and the negative predicted (NPV) value by 10% and 27%, respectively, compared to histopathology alone, thus more accurately identifying patients at high risk [9]. *Starticyte™* is first in its class, however, there is limited data regarding its effectiveness, potential use in clinical practice, and costing estimates.

Accurate predictions of true oral cancer could save lives, reduce morbidity with less traumatic surgeries, increase the duration of productive work lives, and save healthcare costs [10, 11]. Support for its adoption rests on demonstrating value for money, as *Straticyte™* will require an investment by private sectors, since public payers do not cover it. Based on the CEA, the manufacturer, healthcare system, and individual patient will be informed whether investing in this product is worthwhile. The aim of this study is to conduct an early CEA of adding *Straticyte™* to the current standard of care for diagnosing malignant oral lesions in adults.

## Methods

**Fig. 1:**
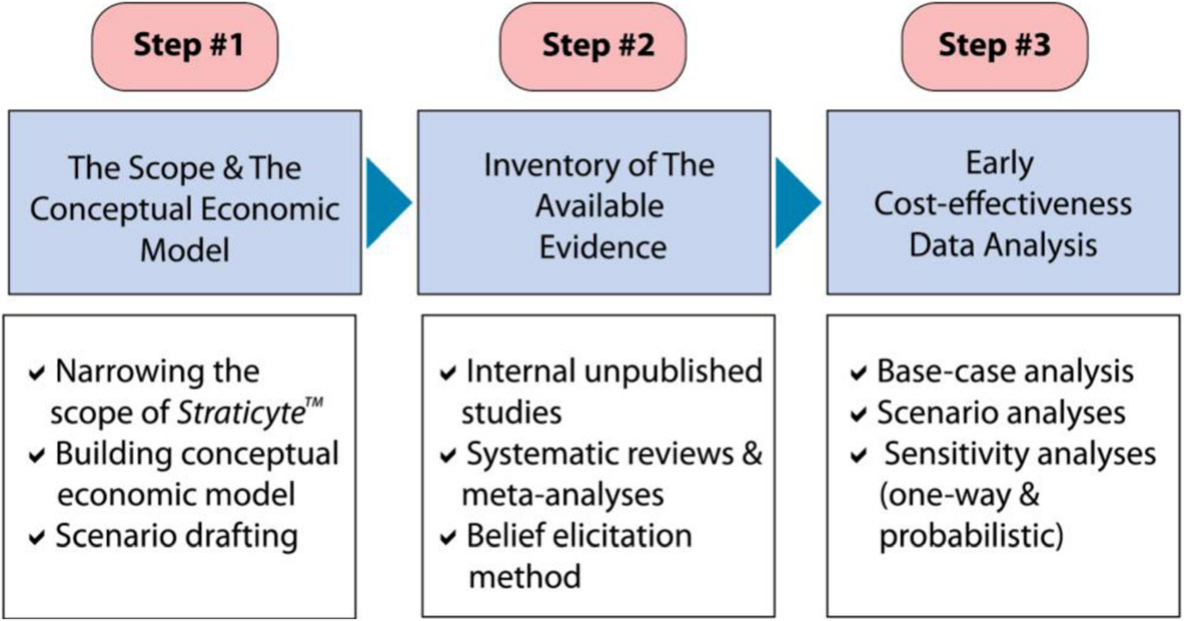
The three key steps followed to conduct early cost-effectiveness analysis. Step#1:Narowing the scope of the economic evaluation, building a conceptual economic model and drafting scenarios for the new prognostic tool (i.e. Straticyte™); Step #2: Inventory of available evidence from internal unpublished resources, systematic review and meta-analysis as well as by utilizing belief elicitation methods to gather scarce evidence where necessary; Step#3: Determining the cost-effectiveness of the Straticyte™ by conducting a base case analysis, scenario analyses and sensitivity analyses (at least deterministic given scarce evidence)

The development of the economic model to determine the cost-effectiveness of *Straticyte™* is summarized in Fig. 1 and described below.

### Step #1: Scope, conceptual economic model and scenario drafting

#### Scope

The potential application of *Starticyte™* in the healthcare system has been assessed through a comprehensive literature search and discussions with test developers, clinicians, and experts in the field of oral cancer. Using this information and the limited available evidence on *Straticyte™,* we narrowed the scope of this CEA by defining the Application, Population, Comparator, Outcome, and Intervention (APCOI) (Additional file 1: Table S2) [4]. This CEA was conducted over a time horizon of five-years and from private payer’s and patient’s perspectives, to capture all relevant differences in future direct and indirect costs and outcomes associated with oral pre-cancer.

#### Conceptual economic model

A five-year CEA was conducted using a decision analytic tree to determine whether a prognostic algorithm for oral cancer that includes *Stratictye™* compared to Histopathology alone in Canada is cost-effective. The model was build using Microsoft Excel^®^ based on four key assumptions (Additional file 1: Table S3) and consists of two arms (Additional file 1: Figure S1). The future costs and outcomes that occur beyond one year associated with both arms were discounted at an annual rate of 5% [12].

#### Scenario drafting

*Straticyte™* indication is not yet finalized, different application paths for *Straticyte™* are possible, hence scenario drafting [13] was used to assess the dynamic aspects of this health technology. In addition to the base case analysis, the effect, cost and cost-effectiveness of two additional possible scenarios where *“Straticyte™”* can successfully be applied were also explored.

### Step #2: Inventory of available evidence and additional data collection on histopathology and *Straticyte™*

The model parameters in Table 1 were gathered from published clinical and economic literature, grey literature, and expert opinion.

**Table 1:** The model input parameters

Parameters^a^	Base case	Deterministic		Probabilistic	Reference/ Sources
		Low value	High value	Distribution	
Transition probabilities^b^					
pSevere	0.271	0.187	0.355	Dirichlet (α_1_ = 29, α_2_ = 78)	12
pModerate	0.355	0.264	0.446	Dirichlet (α_1_ = 38, α_2_ = 69)	12
pMild	0.374	0.282	0.466	Dirichlet (α_1_ = 40, α_2_ = 67)	12
pSevere_C	0.759	0.603	0.914	Beta (α = 22, β = 7)	12
pModerate_C	0.632	0.478	0.785	Beta (α = 24, β = 14)	12
pMild_C	0.375	0.225	0.525	Beta (α = 15, β = 25)	12
pSevere_HighR	0.931	0.839	1.023	Dirichlet (α_1_ = 27, α_2_ = 2)	12
pModerate_HighR	0.158	0.042	0.274	Dirichlet (α_1_ = 6, α_2_ = 32)	12
pMild_HighR	N/A	N/A	N/A	Dirichlet (α_1_ = 0, α_2_ = 40)	12
pSevere_MediumR	0.069	0.000	0.161	Dirichlet (α_1_ = 2, α_2_ = 27)	12
pModerate_MediumR	0.842	0.726	0.958	Dirichlet (α_1_ = 32, α_2_ = 6)	12
pMild_MediumR	0.500	0.345	0.655	Dirichlet (α_1_ = 20, α_2_ = 20)	12
pSevere_LowR	N/A	N/A	N/A	Dirichlet (α_1_ = 0, α_2_ = 29)	12
pModerate_LowR	N/A	N/A	N/A	Dirichlet (α_1_ = 0, α_2_ = 38)	12
pMild_LowR	0.500	0.345	0.655	Dirichlet (α_1_ = 20, α_2_ = 20)	12
pSevere_HighR_C	0.815	0.668	0.961	Beta (α = 22, β = 5)	12
pModerate_HighR_C	0.833	0.535	1.132	Beta (α = 5, β = 1)	12
pMild_HighR_C	N/A	N/A	N/A	Beta (α = 0, β = 0)	12
pSevere_MediumR_C	N/A	N/A	N/A	Beta (α = 0, β = 2)	12
pModerate_MediumR_C	0.594	0.424	0.764	Beta (α = 19, β = 13)	12
pMild_MediumR_C	0.550	0.332	0.768	Beta (α = 11, β = 9)	12
pSevere_LowR_C	N/A	N/A	N/A	Beta (α = 0, β = 0)	12
pModerate_LowR_C	N/A	N/A	N/A	Beta (α = 0, β = 0)	12
pMild_LowR_C	0.200	0.025	0.375	Beta (α = 4, β = 16)	12
Relative risk of developing cancer with Excision (i.e. surgery)					
rrMT	0.51	0.230	1.140	LogNormal (ln (mean = −0.673, SE = 0.408)	SR/MA
Costs and Resources					
cHistopathology	$ 88	$ 70.4	$ 105.6	Gamma (α100=, β = 0.88)	34
cBiomarker	$ 250	$ 200	$ 300	Gamma (α = 100, β = 2.5)	Manufacturer
cExcision	$ 384	$ 307.2	$ 460.8	Gamma (α = 100, β = 3.84)	34
cFollow-up	$ 129	$ 103.2	$ 154.8	Gamma (α = 100, β = 1.29)	34
cPathology	$ 95	$ 76	$ 114	Gamma (α = 100, β = 0.95)	Experts Opinion
cPainMed_T2	$ 12.65	$ 10.15	$ 15.15	Gamma (α = 100, β = 0.127)	Experts Opinion
cPainMed_P	$ 25.17	$ 22.67	$ 27.67	Gamma (α = 100, β = 0.252)	Experts Opinion
cWork_Loss	25.42	20.336	30.504	Gamma (α = 100, β = 0.254)	16
cTransportation	0.575	0.46	0.69	Gamma (α = 100, β = 0.00575)	16
cParking	20 times	16 times	24 times	Gamma (α = 100, β = 0.2)	Assumption
HRSofWORK	24 h	0 h	40 h	Gamma (α = 100, β = 0.240)	Experts Opinion
avgDISTANCE	60 Km	48 Km	72 Km	Gamma (α = 100, β = 0.600)	Assumption
employed	0.9271	0	0	None	16
V_E6M_year	2 visits	1 visits	3 visits	None	Experts Opinion
V_E3M_year	4 visits	3 visits	5 visits	None	Experts Opinion

*p* probability, *C* cancer, *R* risk, *rrMT* relative risk of malignant transformation, *c* cost, *T2* Tylenol 2, *p* peridex, *V* visits, *E6M* every 6 months, *E3M* every 3 months, *SR/MA* systematic review and meta-analysis, *Beta* Beta distribution, *Gamma* Gamma distribution, *Dirichlet* Dirichlet distribution

^a^All the parameters are defined in Additional file 1: Table S9

^b^All transition probabilities are “five year probabilities” and all transition probabilities to cancer are probabilities in the absence of excision (i.e. surgery)

N/A: not available (i.e. there have been no observations in the retrospective study with this outcome)^12^

#### Probabilities

The data used in this model was derived from a retrospective study of 107 cases of dysplasia in Canada [14]. Oral dysplasia biopsy samples were assembled from archives of an oral pathology laboratory [14]. All subjects with histopathological evidence of dysplasia and follow-up information for at least five-years were included. The two primary clinical outcomes were dysplasia progression to cancer, and time in months of dysplasia progression to cancer. This cancer cases were outcomes from patients who have not undergone excision (i.e. surgery) [14]. The uncertainty in probabilities of going from one health state to another was modeled using both Dirichlet and Beta distribution for the purpose of probabilistic sensitivity analysis (PSA) [15]. Where there was a count of zero cancer cases we did not sample from the Dirichlet distribution, instead we assumed constant zero. This was done since there was no information (i.e. observation) on the probability of developing oral cancer in the retrospective study [14].

#### Relative risk (RR) of malignant transformation

To inform the parameter of RR of developing cancer given treatment modality (i.e. relative risk of developing cancer given patients have undergone excision vs. no excision), we conducted a comprehensive systematic literature search to identify clinical studies that investigated the malignant transformation rate (MTR) given treatment modality (Additional file 1: Table S3). The MTRs from the included studies were pooled and the RR of malignant transformation over 5 years was determined using the Cochrane Collaboration Review Manager analysis version 5.2 Statistical Software (RevMan 5.2). The methodology and detailed results of this review can be found in Additional file 1: Figure S2, S3 and Additional file 1: Table S4, S5.

#### Clinical practice by oral and maxillofacial (O&M) surgeons

The belief elicitation method was used to determine the potential impact of *Straticyte™* on clinical practice [16]. Our objective was to determine how O&M surgeons would treat patients with oral dysplasia given the results from *Straticyte™ and* histopathology versus histopathology alone. Questionnaires (Additional file 1: Table S5) were administered face-to-face, requiring 15–30 min to complete, to four O&M surgeons with a minimum of five years of experience in treating patients with oral pre-cancerous lesions (Additional file 1: Table S6). A standardized script was used, explaining the process and the purpose. Questions were prepared with the help of a clinician, and clarified with participants. The outcomes of the elicitation (Additional file 1: Table S7) dictated where in the decision tree (i.e. which branch) the RR of developing oral cancer given excision and the associated costs and resources are applied.

#### Costs and resources

The costs and resource utilizations were gathered from several sources (Table 1). All costs are reported in 2014 CAD, and, if necessary, were corrected by the Canadian consumer price inflation index using the Bank of Canada online inflation calculator [17]. The direct costs associated with the intervention and illness included in this CEA was as follows: oral biopsy (excision, following-up patients), pathology (technician, preparation of report), *Straticyte™* (running the test, technician, reporting the outcome, administrative cost of O&M surgeon and pathologist), pain medication, and gingivitis treatment (Additional file 1: Table S8). The main indirect costs that were included in this CEA were the costs associated with absenteeism from work and transportation costs, included the cost of travel and parking [18, 19].

### Step #3: Early cost-effectiveness data analysis

#### Base case and exploratory scenario analyses

CEAs were conducted in both base-case and scenario cases. This CEA investigated the costs associated with cancer cases avoided. The incremental cost is compared to the incremental health effects [20]. In the base-case scenario, this was the number of cancer cases avoided given the application of *Straticyte™* to all three categories (i.e. Severe, Moderate, Mild) classified by histopathology. In addition, we explored the effect, cost, and cost-effectiveness of two alternative scenarios where *“Straticyte™”* can be applied. For exploratory scenario #1, we examined the number of cancer cases avoided when *Straticyte™* was applied to two categories, moderate and mild cases, and for exploratory scenario #2, we examined cases avoided when *Straticyte™* was applied to only mild cases.

#### Sensitivity analyses

To explore the uncertainty around parameters in the model to find the inputs with the largest impact on the model outcome, one-way sensitivity analyses (OWSA) and probabilistic sensitivity analyses (PSA) were conducted [15]. OWSA provides insight into alternative values for specific parameters that could make a meaningful impact on the model outcome and on the potential decision based upon it. Given this, OWSA was conducted for some of the fixed parameters such as the discount rate, number of follow-ups in a year. The upper and lower values for all included parameters were obtained from published literature. If not available, the mean ± 20% was considered a reasonable range to evaluate a model parameter in the deterministic model. Furthermore, PSA was conducted to take account the overall uncertainty from the combined variability of several factors. A Monte Carlo (MC) simulation method was used to compute the results [15]. A total of 5000 simulations were completed given the fact that early CEAs have an additional level of uncertainty due to limited evidence on *Straticyte™* [15]. Additionally, the collective uncertainty of all of the parameters serves to generate uncertainty at the decision making level. Hence, the net monetary benefit (NMB) approach was used to characterize the decision uncertainty and results presented in a cost-effectiveness acceptability curve (CEAC) [15].

## Results

### Base-case analysis

The incorporation of *Straticyte™* into the current prognostic algorithm (i.e. histopathology) was cost saving as it led to a slightly lower per patient cost and fewer cancer cases over a five-year time horizon compared to histopathology alone (3,360 versus 3,553, and 31 versus 36 per 100 patients, respectively) (Table 2). The histopathology and *Straticyte™* prognostic algorithm was determined to be the dominant strategy (more effective and less costly).

**Table 2:** The incremental cost-effectiveness results from the private and patient’s perspective and time horizon of 5 years

	Histopathology + Stratictye™	Histopathology
Total cost	$ 3,359.62	$ 3,553.28
Total cancer cases	0.31 (31 per 100 patient)	0.36 (36 per 100 patient)
Incremental cost	($ 194.36)	*Histopathology + Stratictye™ DOMINATES Histopathology*
Cancer cases avoided	0.05	
ICER	Dominant	

*ICER:* Incremental cost effectiveness ratio

### Exploratory scenario analyses

Given that *Straticyte™* is not in the market place yet and its indication is not finalized, its cost-effectiveness was assessed when it was only applied to moderate and mild cases (scenario #1) (Table 3). The incorporation of *Straticyte™* remained the dominant strategy in scenario #1 (3,192 versus 3,551, and 28 cancer cases versus 35 cancer cases per 100 patients *Straticyte™* and histopathology versus histopathology alone, respectively). However, when *Straticyte™* was only used for cancer cases (i.e. scenario #2), it no longer was the dominant strategy. Over a five-year time horizon, *Straticyte™* and histopathology was the more expensive approach albeit still more effective than histopathology alone for an ICER of $8610/cancer cases avoided (Table 3).

**Table 3:** The incremental cost-effectiveness results of the exploratory scenarios from the private and patient’s perspective and time horizon of 5 years

	Histopathology + Stratictye™	Histopathology
(A) Scenario #1		
Total cost	$3,192	$3,550.69
Total cancer cases	0.28 (28 per 100 patient	0.35 (35 per 100 patient)
Incremental cost	($ 359)	*Histopathology + Stratictye™ DOMINATES Histopathology*
Cancer cases avoided	0.07	
ICER	Dominates (cost saving)	
(B) Scenario #2		
Total cost	$2,605	$1,399.45
Total cancer cases	0.24 (24 per 100 patient)	0.38 (38 per 100 patient)
Incremental cost	($ 1205)	
Cancer cases avoided	0.14	
ICER	$8,610/ cancer case avoided	

*ICER:* Incremental cost effectiveness ratio

### Sensitivity analyses

#### One-way sensitivity analysis

In almost all cases explored in the OWSA, *Straticyte™* and histopathology was cost saving (more effective and cheaper) compared to histopathology alone. Changes in several parameters, such as the number of visits per year specifically, by applying only 2 visits per year (i.e. every 6 months instead of 3 to the moderate group in histopathology group), relative risk of malignant transformation and probability of developing cancer from mild dysplasia, were found to have meaningful impact on the model outcome. In all three of these cases, the incorporation of *Straticyte™* was associated with slightly higher costs but still better outcomes than histopathology alone.

#### Probabilistic sensitivity analysis

**Fig. 2:**
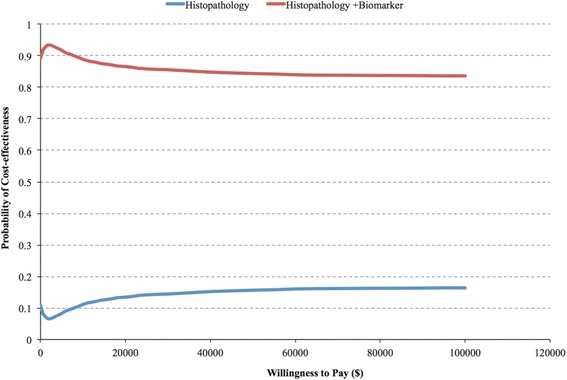
The cost-effectiveness acceptability curve (CEAC) of the early economic evaluation of the new prognostic tool, Straticyte™. Net monetary benefit is used to determine which treatment was cost-effective for each simulation at different willingness to pay thresholds (WTPs) for cancer cases avoided given the use of *Staricyte™* (i.e. biomarker) in combination with the standard of care (i.e. Histopathology)

The CEAC was constructed using MC simulation to demonstrate decision uncertainty. In this study, the CEAC explored the probability of *Straticyte™* and histopathology having the greatest net benefit compared to histopathology alone over a range of potential willingness to pay (WTP) thresholds (Fig. 2). At the lowest WTP threshold, *Straticyte™* and histopathology was the more cost-effective strategy (89% of the simulations) than histopathology alone (11% of the stimulations). With higher thresholds, the probability in which *Straticyte™* and histopathology was the cost-effective option (i.e. the most attractive option) decreased slightly reaching a horizontal asymptote, whereby it offered the highest net benefit in 84% of the simulations (Fig. 2).

## Discussion

### Principal findings

In the base case analysis from the private payers and patient's perspectives, the algorithm of *Straticyte™* and histopathology dominated the current standard of care (SOC), by incurring lower cost and less cancer cases developed over five-years. Uncertainty was considered in this economic model through several sensitivity analyses, for which the results remained robust. The majority of ICER values obtained from all investigated parameters kept the algorithm with *Straticyte™* the dominant strategy, suggesting that it leads to better outcomes and is less expensive than current practices. The model parameters, number of visits per year, relative risk of MT, and probability of developing cancer from mild dysplasia led to less cancer cases, though was slightly more expensive. However, the cost-difference was less than $10,000/QALY, which is substantially lower than the commonly quoted Canadian threshold of $100,000/QALY for the field of oncology, thus remains cost-effective. PSA allowed us to determine the overall impact of the model inputs on the outcome of interest. The result obtained from this analysis was very close to the base-case analysis, where the algorithm with *Straticyte™* was the dominant approach. The CEAC curve generated from the MC simulation demonstrated that the algorithm with *Straticyte™* always had a higher probability of being cost-effective. However, the curve illustrates that there is a slight gap in the available evidence to inform decision-makers to adopt the new technology, since it had less than 100% probability of being cost-effective at very high WTP thresholds. This is not surprising given that *Straticyte™* data is currently limited. As more information is gathered and estimates become more precise, they would progressively fill in this gap, allowing for continuous reassessment and strengthening of the economic output of the model.

### Study in context of relevant literature

There have been no previous CEA of *Straticyte™* and literature on early CEA is limited. Recently, a few studies have presented general overview of methods to conduct early CEA and briefly applied suggested methods into the process of late CEA to demonstrate their potential usefulness in conducting early CEA [3, 5, 13, 16, 21–24]. The literature highlights that integration of health economic modeling into early decision is not extensively practiced in pharmaceutical industry, and nearly absent for devices [25]. In pharmaceutical companies, CEAs are mostly conducted for marketing and reimbursement purposes versus research and development, despite the fact that economic factors are usually considered the second leading cause for research termination of an early technology [26, 27].

### Limitations and strengths

This early CEA is associated with several limitations. First, there is a paucity of high level clinical evidence regarding the effectiveness of *Straticyte™*, which is the nature of conducting any kind of analysis at a product’s early stages of development [4]. We attempted to account for this by conducting several sensitivity analyses to test our assumptions of effectiveness and clinical use. Second, methods used in this early economic analysis are vaguely described in the literature and are commonly only pilot studies [3, 13, 16, 21–24]. Given the nature of this analysis, these methods can be conceptually challenging and rely highly on a number of assumptions [28, 29]. This makes the results very susceptible to critique by experts in the field pertaining to the technology, despite attempts to account for these assumptions through sensitivity analyses. To ensure clinical relevance, we sought expert advice throughout the evaluation process to help identify gaps and provide direction. Lastly, since some of the information such as the frequency of follow-ups was inputs by experts’ opinion based on their everyday practice, stricter follow-up (3 months vs. 6 months) could potential be more effective over long-term in identifying new pre-cancerous lesions, recurrences, which may have resulted in improved outcomes due to earlier treatment. Therefore, another major limitation of this study was not considering the potential additional benefits due to stricter follow-up by O&M surgeons. This paper has several strengths. First, we conducted an extensive review of the literature to identify methodologies of early CEA (Khoudigian-Sinani et al. manuscript in preparation). Second, we sought clinical expert opinions as well as opinions of leaders in HTA to inform our analyses. Third, we are the first to incorporate multiple methods that were suggested and piloted in the literature to complete a thorough early CEA to determine the potential value of *Straticyte™.*

### Implications for clinicians and policymakers

The considerable burden of disease and expense of oral cancer in Canada highlights the importance of accurately predicting the risk of developing oral cancer to both patients and the health care system [5]. *Straticyte™,* is at its early pre-market stage of its lifecycle, hence this was an attempt to compare the costs and outcomes of incorporating it to the current prognostic algorithm using limited data related to its clinical use and effectiveness. Decision analytical modeling techniques as well as qualitative methods, such as belief elicitation method and scenario drafting, were applied, and parameters for which the model outcome is most sensitive was explored. This provides a thorough early CEA that is important for clinicians and policymakers to consider. Furthermore, whilst presenting a successful attempt in early modeling and the difficulties associated with it, this paper creates a potential foundation to work on and build a guiding framework in creating more robust early models, with useful insight into the potential value of the product at that moment as well as meet the requirements of fully developed models at late stages of the product’s life cycle.

### Unanswered questions and future research considerations

Canadian policy makers have to make informed decisions on how to allocate resources for the population in the most efficient manner, given increasing health expenditures and scarce resources [1]. These decisions generally are based on both clinical and cost-effectiveness evidence of new health technologies compared with standard care or alternative technologies [1]. Even though cost-effectiveness analysis within health technology assessment has long been recognized as a compelling way to ascertain value for buyers, its role in the allocation of research and development by companies is not well described. There is no set guideline that helps guide on how to conduct CEAs during the early stages of a technology’s development life cycle and how to deal with challenges associated with the lack of both clinical and economic evidence. Despite the development in health economic methods to support reimbursement after the product is in the market place, the use of CEA at the early stages of product’s development is less explored and needs further research.

## Conclusion

This early CEA demonstrates a high probability of success that *Straticyte™* will be cost-effective. This supports continued investment by the manufacturer, and that investment by the healthcare system and individual patients may be worthwhile. Data is currently limited, and as the product cycle progresses, additional information will inform the model and provide more accurate estimates of the technology’s cost effectiveness.

## Additional file

Additional file 1: Figure S1. The Decision Analytic Model for Oral Pre-cancerous Lesions. Patients who have already undergone biopsy are diagnosed either by histopathology, where the dysplasia is graded as severe, moderate or mild based on the extent of the architectural and cytological changes, or with histopathology and *Stratictye™*, where patients in each dysplasia grading are further classified as high, medium, or low risk of developing oral cancer based on the result of the *Stratictye™* test. These categorizations are mapped in mutually exclusive pathways. **Table S1.** Qualitative and Quantitative Approaches to Conduct Early CEA. **Table S2.** Defining the Scope of Early Cost-effectiveness (CEA) Model. **Table S3.** Four Key Model Assumptions. **Table S4.** The Literature Search Strategy. **Figure S2.** The PRISMA flow chart. **Table S5.** The characteristics of the included studies [30–34]. **Figure S3.** The forest plot by RevMan [35]. **Table S6.** Questionnaire for Oral and Maxillofacial Surgeons. **Table S7.** The outcome of the Questionnaires. **Table S8.** Costing Details [36]. **Table S9.** Definitions of the model input parameters. **Figure S4.** The scattered plot of 5000 Monte Carlo simulations. (DOCX 2425 kb)

## Abbreviations

APCOI: Application, population, comparator, outcome, intervention
CAD: Canadian
CEA: Cost-effectiveness analysis
CEAC: Cost-effectiveness acceptability curve
EE: Economic evaluation
ICER: Incremental cost-effectiveness ratio
MC: Monte Carlo
MT: Malignant transformation
MTR: Malignant transformation rate
NMB: Net monetary benefit
NPV: Negative predictive value
O&M: Oral and maxillofacial
OWSA: One-way sensitivity analysis
PPV: Positive predictive value
PSA: Probabilistic sensitivity analysis
QALY: Quality adjusted life years
RR: Relative risk
WTP: Willingness to pay threshold
