# Supplementary material for: The premarket assessment of the cost-effectiveness of a predictive technology “Straticyte™” for the early detection of oral cancer: a decision analytic model
Source: Health Econ Rev. 2017 Oct 2;7:35. doi: 10.1186/s13561-017-0170-6 (PMC5624864; doi:10.1186/s13561-017-0170-6)
Supplement: Additional file 1: Figure S1. — The Decision Analytic Model for Oral Pre-cancerous Lesions. Patients who have already undergone biopsy are diagnosed either by histopathology, where the dysplasia is graded as severe, moderate or mild based on the extent of the architectural and cytological changes, or with histopathology and Stratictye™, where patients in each dysplasia grading are further classified as high, medium, or low risk of developing oral cancer based on the result of the Stratictye™ test. These categorizations are mapped in mutually exclusive pathways. Table S1. Qualitative and Quantitative Approaches to Conduct Early CEA. Table S2. Defining the Scope of Early Cost-effectiveness (CEA) Model. Table S3. Four Key Model Assumptions. Table S4. The Literature Search Strategy. Figure S2. The PRISMA flow chart. Table S5. The characteristics of the included studies [30–34]. Figure S3. The forest plot by RevMan [35]. Table S6. Questionnaire for Oral and Maxillofacial Surgeons. Table S7. The outcome of the Questionnaires. Table S8. Costing Details [36]. Table S9. Definitions of the model input parameters. Figure S4. The scattered plot of 5000 Monte Carlo simulations. (DOCX 2425 kb) [file 13561_2017_170_MOESM1_ESM.docx]

***The Premarket Assessment of the Cost-effectiveness of a Predictive Technology “Straticyte^TM^” for the Early Detection of Oral Cancer: A decision analytic model***

**Supplementary Appendix**

Appendix Figure 1


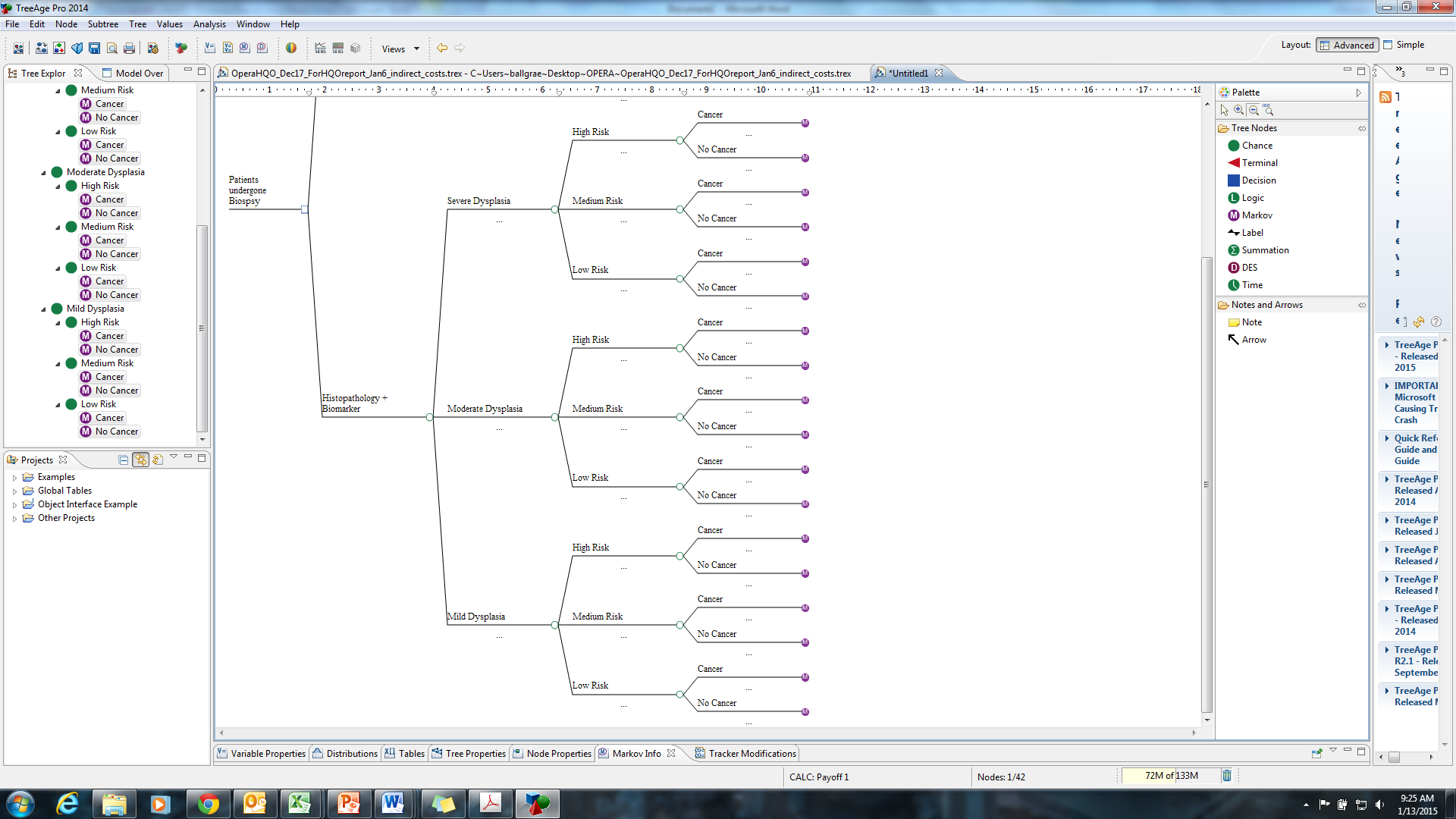

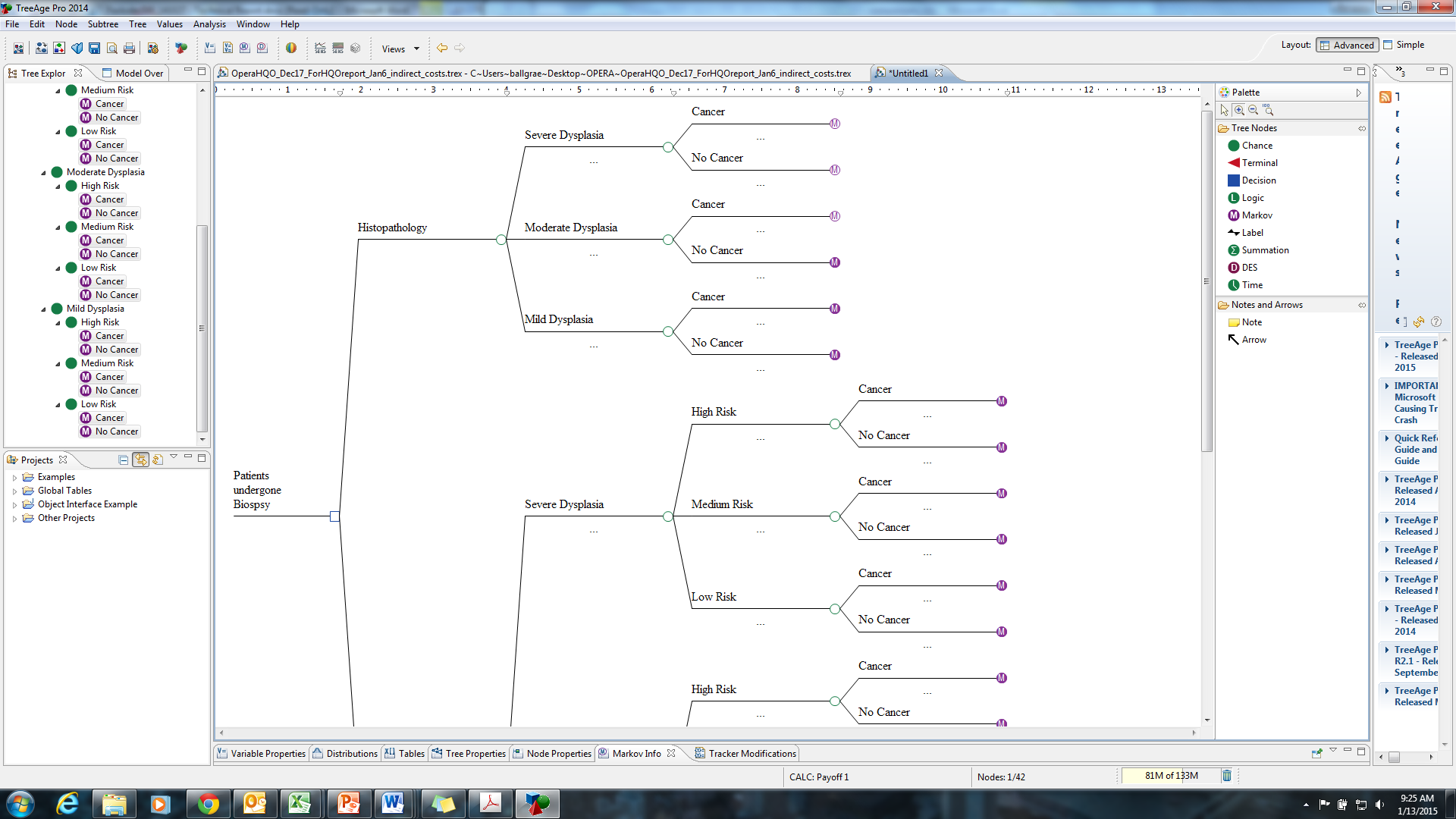


Figure 1: The Decision Analytic Model for Oral Pre-cancerous Lesions. Patients who have already undergone biopsy are diagnosed either by histopathology, where the dysplasia is graded as severe, moderate or mild based on the extent of the architectural and cytological changes, or with histopathology and *Stratictye^TM^*, where patients in each dysplasia grading are further classified as high, medium, or low risk of developing oral cancer based on the result of the *Stratictye^TM^* test. These categorizations are mapped in mutually exclusive pathways.

Appendix Table 1

Table 1: Qualitative and Quantitative Approaches to Conduct Early CEA

| A. Quantitative Approaches: |
| --- |
| 1. Scenario Drafting |
| 2. Belief Elicitation Method |
| B. Qualitative Approaches (mostly used for drugs): |
| 1. Headroom Analysis |
| 2. Bayesian Analysis |
| 3. Value of Information Analysis |

Appendix Table 2

Table 2: Defining the Scope of Early Cost-effectiveness (CEA) Model

| Application: | The application was using “Straticyte^TM^” in the health-care system to predict the risk of developing oral cancer for patients with pre-malignant lesions |
| --- | --- |
| Population: | The target population was individuals 35 years of age and older and have undergone biopsy for suspected oral cancer |
| Comparator: | The comparator was the current prognostic test, histopathology (i.e. gold standard), applied by oral and maxillofacial (O&M) surgeons |
| Outcome: | The outcomes taken into account were effectiveness of “Straticyte^TM^”, defined as cancer cases avoided, as well as the direct and indirect costs |
| Intervention: | The intervention was the new prognostic strategy, “Starticyte^TM^”, in addition to Histopathology (i.e. gold standard) |

Appendix Table 3

Table 3: Four Key Model Assumptions

| 1. Patients had dysplasia and were not treated and that the malignant transformation rate observed, reflects the natural disease progression. |
| --- |
| 2. Treatment decision depending on Straticyte^TM +^ Histopathology and histopathology alone were based on expert opinion. |
| 3. The number of days off work (on average 3 days, ranges from 0 to 7 days) after excision was based on expert opinion. |
| 4. The most common medications prescribed to patients who have undergone excision were Tylenol 2 and Peridex, which was based on expert opinion. |

Appendix Table 4

Table 4: The Literature Search Strategy

| **OVERVIEW** |  | |
| --- | --- | --- |
| Interface: | OVID | |
| Databases: | MEDLINE(R) In-Process & Other Non-Indexed Citations and Ovid MEDLINE(R)*1946 to Present* | |
| Date of Search: | November 11^th^, 2014 | |
| Updated Search: | May 9^th^, 2016 | |
| Study Types: | Randomized controlled trials; controlled clinical trials; multicenter studies; cohort studies; case control studies; observational studies | |
| Limits: | None | |
| **SYNTAX GUIDE** | |  |
| .mp | Title, abstract, original title, name of substance word, subject heading word, keyword heading word, protocol supplementary concept word, rare disease supplementary concept word, unique identifier | |

| **Database Strategy** | | |
| --- | --- | --- |
| **#** | **Searches** | **Results** |
| 1 | Dysplasia.mp | 61545 |
| 2 | Oral.mp | 528729 |
| 3 | 1 or 2 | 2981 |
| 4 | Mouth.mp | 126950 |
| 5 | 1 or 4 | 1690 |
| 6 | 3 or 5 | 3274 |
| 7 | (Progression or follow-up or follow up or treatment or cohort or natural history or recurrence).mp | 4549690 |
| 8 | 6 and 7 | 1189 |

| **OVERVIEW** | |  | |
| --- | --- | --- | --- |
| Interface: | | OVID | |
| Databases: | | EMBASE 1974 to 2014 November 06 | |
| Date of Search: | | November 11^th^, 2014 | |
| Updated Search: | | May 9^th^, 2016 | |
| Study Types: | | Randomized controlled trials; controlled clinical trials; multicenter studies; cohort studies; case control studies; observational studies | |
| Limits: | | None | |
| **SYNTAX GUIDE** | | |  |
| .mp | Title, abstract, original title, name of substance word, subject heading word, keyword heading word, protocol supplementary concept word, rare disease supplementary concept word, unique identifier | | |

| **Database Strategy** | | |
| --- | --- | --- |
| **#** | **Searches** | **Results** |
| 1 | Dysplasia.mp | 92591 |
| 2 | Oral.mp | 905041 |
| 3 | 1 or 2 | 4304 |
| 4 | Mouth.mp | 195526 |
| 5 | 1 or 4 | 2664 |
| 6 | 3 or 5 | 5040 |
| 7 | (Progression or follow-up or follow up or treatment or cohort or natural history or recurrence).mp | 5792535 |
| 8 | 6 and 7 | 1480 |

Appendix Figure 2

EMBASE
(n = 1480)

2669 Total Records

1036 duplicates excluded

1633 Records
(title and abstract)
screened

1573 Records excluded

60 Full-text articles assessed for
eligibility

55 Studies excluded:

- Not RCT or OB (n=21)
- No comparator (n= 25)
- Wrong outcome (n=5)
- Not English (n=4)

5 Studies included in synthesis

Identification

Screening

Eligibility

MEDLINE
(n = 1189)

Included

1 grey literature

Figure 2: The PRISMA flow chart

Appendix Table 5

Table 5: The characteristics of the included studies

| Author,  year | Country | Methodology/ setting | Date of enrollment | Mean age | # of cancer cases/ total # of surgically treated patients | # of cancer cases/ total # of non-surgically treated patients |
| --- | --- | --- | --- | --- | --- | --- |
| Saito,  2001 [28] | Japan | Retrospective/ Hospital | 1976-1997 | 54 | 5/91 | 4/51 |
| Banoczy,  1976 [29] | Hungary | Retrospective/ Hospital | NR | NR | 1/44 | 8/15 |
| Arduino,  2009 [30] | Italy | Retrospective/ Hospital | 1991-2007 | 63.58 | 12/133 | 3/74 |
| Arnaoutakis,  2013 [31] | USA | Retrospective/ Hospital | 1990-2011 | 59.2 | 14/75 | 4/51 |
| Holmstrup,  2006 [32] | Denmark | Retrospective/ Pathology laboratory | 1977-1997 | 60.8 | 6/67 | 2/21 |

Appendix Figure 3


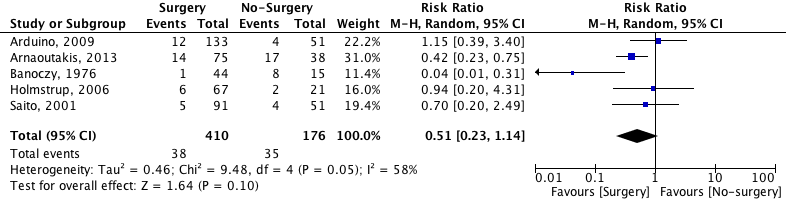


Figure 3: The forest plot by RevMan [33]

***Brief description of how RR is estimated and used in this economic evaluation****: The MTRs from the included studies were pooled and the RR of malignant transformation over 5 years was determined using the Cochrane Collaboration Review Manager analysis version 5.2 Statistical Software (RevMan 5.2). Following this, the outcome of elicitation (details found in the “Clinical practice by oral and maxillofacial (O&M) surgeons” section of the manuscript on pages 3-4 as well as Appendix Table 7) dictated where in the decision tree (i.e. which branch) the RR of developing oral cancer given excision and the associated costs and resources are applied.*

Appendix Table 6

Table 6: Questionnaire for Oral and Maxillofacial Surgeons

|  | |  | | |  | | **Date of interview:** | | | | |  | |
| --- | --- | --- | --- | --- | --- | --- | --- | --- | --- | --- | --- | --- | --- |
| **Interviewer information** | | | | |  | |  | | |  | |  | |
| Name: | | |  | | | | | | | | |  | |
| **Interviewee Information** | | | | |  | |  | | |  | |  | |
| Name: | | |  | | | | | | | | |  | |
| Occupation: | | |  | | | | | | | | |  | |
| Address: | | |  | | | | | | | | |  | |
|  | | |  | | | | | | | | |  | |
|  | | |  | | | | | | | | |  | |
| Phone: | | |  | | | | | | | | |  | |
| E-mail: | | |  | | | | | | | | |  | |
|  | |  | | |  | |  | | |  | |  | |
| **PROJECT TITLE:** | | | | | | |  | | |  | |  | |
|  | | | | | | | | | | | | | |
|  | |  | | |  | |  | | |  | |  | |
| **PROJECT OVERVIEW:** | | | | |  | |  | | |  | |  | |
| *Health economic evaluation provides information about the value for money of new healthcare technologies, and is increasingly used to guide the allocation of scarce resources based on maximizing health gain. The molecular diagnostics company, PDI, has developed a technology to improve the identification of patients at high risk for oral cancers for early intervention, and distinguish abnormal cell growth that will not become malignant, compared to the current gold standard alone.  This more accurate diagnosis could save lives, reduce morbidity from traumatic surgeries, increase the duration of productive work lives, and save healthcare costs. The purpose of this internship is to develop a health economic model to evaluate the cost-effectiveness of the technology, as well as its social impact. The results of the model will help determine whether the new technology demonstrates economic value.* | | | | | | | | | | | | | |
|  | | | | | | | | | | | | | |
| **DEFINITIONS:** | | | | | | | |  | | |  | |  |
| *No risk factor: none* | | | | | | | | | | | | | |
| *Moderate risk factor: Only smokes; Only Drinks; HPV/ RBV infected; Immune-compromised; HIV infected* | | | | | | | | | | | | | |
| *High risk factor: Prior Cancer; more than one of these: smoker, alcoholic, HPV/RBV infected, Immune-compromised, HIV infected* | | | | | | | | | | | | | |
|  | | | | |  | | |  | | |  | |  |
|  | | | | | | | | | | | | | |
| **QUESTIONNAIRS** | | | | | |  | | |  | | |  | |
| **Prognostic tool** | | | | **Biomarker** | | **Risk Factor** | | | **Treatment** | | | **Follow-up** | |
| **Histopathology Only** | Severe Dysplasia | | | - | | No Risk Factor | | |  | | |  | |
|  |  |  |  |  |  | Moderate Risk Factor | | |  | | |  |  |
|  |  |  |  |  |  | High Risk Factor | | |  | | |  |  |
|  | Moderate Dysplasia | | | - | | No Risk Factor | | |  | | |  | |
|  |  |  |  |  |  | Moderate Risk Factor | | |  | | |  |  |
|  |  |  |  |  |  | High Risk Factor | | |  | | |  |  |
|  | Mild Dysplasia | | | - | | No Risk Factor | | |  | | |  | |
|  |  |  |  |  |  | Moderate Risk Factor | | |  | | |  |  |
|  |  |  |  |  |  | High Risk Factor | | |  | | |  |  |
| **Histopathology + Biomarker** | Severe Dysplasia | | | High Risk Biomarker | | No Risk Factor | | |  | | |  | |
|  |  |  |  |  |  | Moderate Risk Factor | | |  | | |  |  |
|  |  |  |  |  |  | High Risk Factor | | |  | | |  |  |
|  |  |  |  | Medium Risk Biomarker | | No Risk Factor | | |  | | |  | |
|  |  |  |  |  |  | Moderate Risk Factor | | |  | | |  |  |
|  |  |  |  |  |  | High Risk Factor | | |  | | |  |  |
|  |  |  |  | Low Risk Biomarker | | No Risk Factor | | |  | | |  | |
|  |  |  |  |  |  | Moderate Risk Factor | | |  | | |  |  |
|  |  |  |  |  |  | High Risk Factor | | |  | | |  |  |
|  | Moderate Dysplasia | | | High Risk Biomarker | | No Risk Factor | | |  | | |  | |
|  |  |  |  |  |  | Moderate Risk Factor | | |  | | |  |  |
|  |  |  |  |  |  | High Risk Factor | | |  | | |  |  |
|  |  |  |  | Medium Risk Biomarker | | No Risk Factor | | |  | | |  | |
|  |  |  |  |  |  | Moderate Risk Factor | | |  | | |  |  |
|  |  |  |  |  |  | High Risk Factor | | |  | | |  |  |
|  |  |  |  | Low Risk Biomarker | | No Risk Factor | | |  | | |  | |
|  |  |  |  |  |  | Moderate Risk Factor | | |  | | |  |  |
|  |  |  |  |  |  | High Risk Factor | | |  | | |  |  |
|  | Mild Dysplasia | | | High Risk Biomarker | | No Risk Factor | | |  | | |  | |
|  |  |  |  |  |  | Moderate Risk Factor | | |  | | |  |  |
|  |  |  |  |  |  | High Risk Factor | | |  | | |  |  |
|  |  |  |  | Medium Risk Biomarker | | No Risk Factor | | |  | | |  | |
|  |  |  |  |  |  | Moderate Risk Factor | | |  | | |  |  |
|  |  |  |  |  |  | High Risk Factor | | |  | | |  |  |
|  |  |  |  | Low Risk Biomarker | | No Risk Factor | | |  | | |  | |
|  |  |  |  |  |  | Moderate Risk Factor | | |  | | |  |  |
|  |  |  |  |  |  | High Risk Factor | | |  | | |  |  |

Appendix Table 7

Table 7: The outcome of the Questionnaires

|  | Treatment | Follow-up |
| --- | --- | --- |
| Histopathology | | |
| Severe Dysplasia | Local excision | Every 6 months for 5 years |
| Moderate Dysplasia | Local excision | Every 3 months for 5 years |
| Mild Dysplasia | Monitor | Every 6 months for 2 years |
| Straticyte^TM^  and Histopathology | | |
| Severe Dysplasia + High Risk | Local excision | Every 6 months for 5 years |
| Severe Dysplasia + Medium Risk | Local excision | Every 6 months for 5 years |
| Severe Dysplasia + Low Risk | Local excision | Every 6 months for 5 years |
| Moderate Dysplasia + High Risk | Local excision | Every 6 months for 5 years |
| Moderate Dysplasia + Medium Risk | Local excision | Every 6 months for 5 years |
| Moderate Dysplasia + Low Risk | Local excision | Every 3 months for 5 years |
| Mild Dysplasia + High Risk | Local excision | Every 6 months for 5 years |
| Mild Dysplasia + Medium Risk | Local excision | Every 6 months for 5 years |
| Mild Dysplasia + Low Risk | Monitor | Every 6 months for 2 years |

Appendix Table 8

Table 8: Costing Details [34]

| Oral Biopsy Cost: | The excision, and cost of monitoring patients were estimated from the 2014 Ontario Dental Association (ODA) Suggested Fee Guide For Dental Services |
| --- | --- |
| Cost of Pathology | The technician and preparation of the pathology report, were obtained by interviewing a pathologist from the Mount Sinai Hospital – Pathology and Laboratory Medicine located in Toronto, Canada |
| Cost of *Straticyte^TM^* | The cost of running the test, the technician cost, the cost of reporting the outcome of the test as well as the administrative costs for the O&M surgeon and the pathologist, was derived from Proteocyte Diagnostic Inc |
| Prescribed Drugs | *Tylenol 2^®^* to control pain and *Peridex^TM^* to treat gingivitis were taken into consideration in patients who have undergone excision |

Appendix Table 9

Table 9: Definitions of the model input parameters

| **Table 7:** The definition of the model input parameters | |
| --- | --- |
| **Parameters** | **Definitions** |
| **Transition Probabilities** | |
| pSevere | *Probability of severe cases based on Histopathology over 5 years* |
| pModerate | *Probability of moderate cases based on Histopathology over 5 years* |
| pMild | *Probability of mild cases based on Histopathology over 5 years* |
| pSevere_C | *Probability of patients who developed cancer over 5 years who were diagnosed as severe cases based on Histopathology* |
| pModerate_C | *Probability of patients who developed cancer over 5 years who were diagnosed as moderate cases based on Histopathology* |
| pMild_C | *Probability of patients who developed cancer over 5 years who were diagnosed as mild cases based on Histopathology* |
| pSevere_HighR | *Probability of severe cases based on Histopathology and high risk prognosis with Starticyte^TM^ over 5 years* |
| pModerate_HighR | *Probability of moderate cases based on Histopathology and high risk prognosis with Starticyte^TM^ over 5 years* |
| pMild_HighR | *Probability of mild cases based on Histopathology and high risk prognosis with Starticyte^TM^ over 5 years* |
| pSevere_MediumR | *Probability of severe cases based on Histopathology and medium risk prognosis with Starticyte^TM^ over 5 years* |
| pModerate_MediumR | *Probability of moderate cases based on Histopathology and medium risk prognosis with Starticyte^TM^ over 5 years* |
| pMild_MediumR | *Probability of mild cases based on Histopathology and medium risk prognosis with Starticyte^TM^ over 5 years* |
| pSevere_LowR | *Probability of severe cases based on Histopathology and low risk prognosis with Starticyte^TM^ over 5 years* |
| pModerate_LowR | *Probability of moderate cases based on Histopathology and low risk prognosis with Starticyte^TM^ over 5 years* |
| pMild_LowR | *Probability of Mild cases based on Histopathology and low risk prognosis with Starticyte^TM^ over 5 years* |
| pSevere_HighR_C | *Probability of patients who developed cancer over 5 years who were diagnosed as severe cases based on Histopathology and high risk prognosis with Starticyte^TM^* |
| pModerate_HighR_C | *Probability of patients who developed cancer over 5 years who were diagnosed as moderate cases based on Histopathology and high risk prognosis with Starticyte^TM^* |
| pMild_HighR_C | *Probability of patients who developed cancer over 5 years who were diagnosed as mild cases based on Histopathology and high risk prognosis with Starticyte^TM^* |
| pSevere_MediumR_C | *Probability of patients who developed cancer over 5 years who were diagnosed as severe cases based on Histopathology and medium risk prognosis with Starticyte^TM^* |
| pModerate_MediumR_C | *Probability of patients who developed cancer over 5 years who were diagnosed as moderate cases based on Histopathology and medium risk prognosis with Starticyte^TM^* |
| pMild_MediumR_C | *Probability of patients who developed cancer over 5 years who were diagnosed as mild cases based on Histopathology and medium risk prognosis with Starticyte^TM^* |
| pSevere_LowR_C | *Probability of patients who developed cancer over 5 years who were diagnosed as severe cases based on Histopathology and low risk prognosis with Starticyte^TM^* |
| pModerate_LowR_C | *Probability of patients who developed cancer over 5 years who were diagnosed as moderate cases based on Histopathology and low risk prognosis with Starticyte^TM^* |
| pMild_LowR_C | *Probability of patients who developed cancer over 5 years who were diagnosed as mild cases based on Histopathology and low risk prognosis with Starticyte^TM^* |
| **Malignant Transformation Rate (MTR)** | |
| rrMT | *Relative risk of Malignant Transformation of oral lesions by treatment modality (surgical excision vs. no surgery)* |
| **Costs and Resources** | |
| cHistopathology | *Cost of Biopsy by incision ($73 + 20% higher since oral surgeons do it)* |
| cBiomarker | *Cost of Biomarker test based on Proteocyte Inc.* |
| cExcision | *Average cost of excision (≤1cm, 1-2cm, 2-3cm, 3-4cm, 4-6cm, 6-9cm, 9-15cm, ≥15cm)* |
| cMonitor | *Cost of monioring patients (per visit)* |
| cPathology | *Cost of technician & pathology report* |
| cPainMed_T2 | *Cost of pain medication if prescribed after the excision (Surgery)* |
| cPainMed_P | *Cost of antiseptics if prescribed after the excision (Surgery)* |
| cWork_Loss | *Average hourly wage rate* |
| cTransportation | *Cost of transportation per km travelled by patients have to visit oral surgeon for monitoring* |
| cParking | *Cost of parking when patients have to visit oral surgeon for monitoring* |
| HRSofWORK | *Hours of days of work after the excision* |
| avgDISTANCE | *Average distance a patient has to travel to get to an Oral & Maxillofacial Clinic* |
| employed | *Probability of patients employed in Canada* |
| V_E6M_year | *Number of days they are monitored per year* |
| V_E3M_year | *Number of days they are monitored per year* |
| *p= probability; C= cancer; R= risk; rrMT= relative risk of malignant transformation; c= cost; T2= Tylenol 2; p= peridex; V= visits; E6M= every 6 months; E3M= every 3 months.* | |

Appendix Figure 4


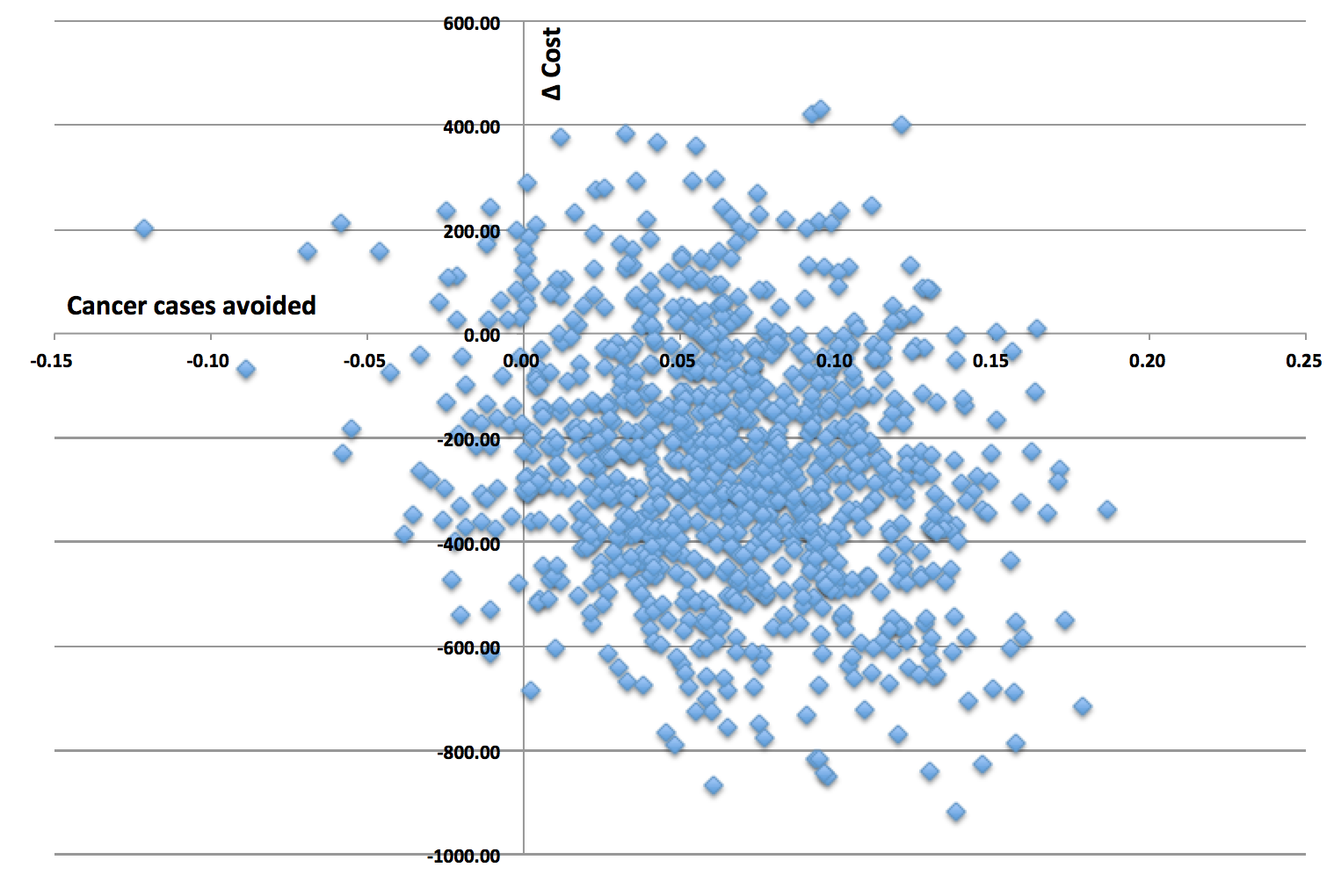


Figure 4: The scattered plot of 5000 Monte Carlo simulations
